# The role of microwave radiation in extractive desulfurization of real diesel fuel for green environment: an experimental and computational investigation

**DOI:** 10.1186/s13065-024-01292-2

**Published:** 2024-10-14

**Authors:** Hamida Y. Mostafa, Ghada E. Khedr, Ard Elshifa M. E. Mohamed, Dina M. Abd El-Aty

**Affiliations:** 1 Refining Division, Egyptian Petroleum Research Institute(EPRI), 1 Ahmed El- Zomor St., Nasr City, 11727 Cairo Egypt; 2 Nanotechnology Center, Central Analytical Laboratories, Egyptian Petroleum Research Institute (EPRI), 1 Ahmed El- Zomor St., Nasr City, 11727 Cairo Egypt; 3 Analysis and Evaluation Department, Egyptian Petroleum Research Institute (EPRI), 1 Ahmed El-Zomor St., Nasr City, 11727 Cairo Egypt; 4 Department of Chemistry, College of Science, Qassim University, Buraydah, Qassim Saudi Arabia; 5 Department of Chemistry, College of Science, Alneelain University, Khartoum, Sudan

**Keywords:** Microwave assisted extraction, Methanol, Acetonitrile, Ethyl acetoacetate, Diesel fuel, Desulfurization

## Abstract

The process of removing sulfur compounds and aromatic compounds to produce clean fuel is an important and effective contribution to the processes of mitigating and adapting to climate change. In contrast, it is necessary to find an innovative way to remove sulfur and carcinogenic aromatic compounds because clean, low-sulfur diesel is commonly used in all countries of the world at the present time. Therefore, in this work, we have studied the effect of the microwave radiation power and the irradiation time with the use of more than one type of organic solvent; methanol, acetonitrile and ethyl acetoacetate; as an extractant and solvent to feed ratio impact on the removal of sulfur and aromatic compounds of a real diesel fuel feed which has 450 ppm sulfur content and 16 wt% aromatic Content. The results showed that the best solvent used during this work was ethyl acetoacetate. According to the results, high sulfur removal (≈ 92%) was accomplished with microwave-assisted extractive desulfurization technique under the following ideal conditions: the irradiation time is 7 min, the solvent feed ratio is 3:1 and the microwave intensity is 180 W. To reveal the mechanism of microwave-assisted extractive desulfurization via different organic solvents, a theoretical study including structural examination and interaction energy analysis on the interaction between dibenzothiophene (DBT) or dimethyl dibenzothiophene (DMDBT) and the different organic solvents was also conducted.

## Introduction

Burning high sulfur content of diesel fuel inside automotive engines produces sulfur oxides fumes and particulate matters that cause acid rains, catalyst deactivation, and corrosion of equipment, environmental pollution and human diseases. Because of these worries, the United States Environmental Protection Agency (US-EPA) has instructed using low-sulfur diesel fuel to mitigate pollution [1]. The conventional desulfurization process was hydro-desulfurization process, in this process large amount of catalyst, high temperature and large amount of hydrogen have been used and the presence of sulfur inside the aromatic compound (as dibenzothiophene or 4,6-dimethyldibenzothiophene) that decrease the efficiency of that process. The traditional solvent extraction process which used in the petroleum refining companies, this process uses thousands of litters from solvents and its efficiency don’t exceed to 49% removal for sulfur compounds [2–4].

So, there is a great effort to use more efficient, and more economically sulfur removal process as, biodegradation of sulfur compound, adsorption using high surface area adsorbent material, oxidative desulfurization process, photo-oxidative desulfurization, ionic-liquid extractive desulfurization, microwave-assisted solvent extraction and ultrasonic-assisted desulfurization. Where in these processes there in hydrogen used, little amount of solvent, little amount of highly efficient catalysts, few minutes, smaller equipment can be used. So, by using these methods there is saving in time and money [5–9].

Microwave-assisted extractive desulfurization is considered as a novel economic technology in fuel refining process [10]. The microwave-assisted solvent extraction is more simple, easy, and the most effective refining process than the conventional processes, where it used to reduce the reaction time and chemical consumption. Additional great benefit is that it is an automatic technique, which makes it reproducible. Moreover, MAE can be used in conjunction with other methods such as pulsed electric fields or ultrasound and is energy-efficient. Nevertheless, MAE is not without its drawbacks. Microwave extraction equipment can have a high initial capital cost, which could restrict its industrial application. Further investigation into the scalability of MAE is also necessary to guarantee consistent outcomes in large-scale operations. Notwithstanding these difficulties, MAE is a potential technique for extraction operations because of its advantages, which include increased efficiency and increased polarity in the extraction media [11].

The microwave heating depends on the conversion of electromagnetic energy from microwave to thermal energy by increasing the agitation of water molecules or the charged ions which exposed to the microwave. The microwave is an electrical equipment that heats the materials by exposing them to electromagnetic radiation which creates from the transmitter that inside the microwave box. The interaction between the electromagnetic radiation and specific materials e.g. solvents depend on the ability of that solvent to convert the electromagnetic radiation to heat and that ability depends on the polarity of the solvent [12, 13]. Where, as increasing the polarity of solvent, increasing the interaction between the electromagnetic field and the polar solvent and increasing the absorption of microwave energy that causes higher rate reaction [14, 15].

So, the solvents that used in microwave applications must be a dipole to be able to generate heat when irradiated by microwave (their molecular structure must be charged by partly positive and partly negative charge). The microwave field is oscillating and the dipoles in the field were aligning to the oscillating field. This alignment reasons rotation, which causes friction and then heat energy [10, 16, 17].

The goal of this research is to introduce a unique approach to diesel fuel desulfurization: the microwave-assisted extractive desulfurization process. Additionally, a locally built extractive desulfurization unit equipped with a microwave generating unit is presented as a novel extraction technology. Comparing the effectiveness of microwave liquid–liquid extraction—a novel method—with the traditional method for the removal of sulfur and aromatics.

We aim to elucidate the underlying mechanisms governing the desulfurization process and complement the experimental work so the computational analysis was performed [18]. We seek to develop more efficient and sustainable desulfurization strategies with broad applications in energy production and environmental remediation [19, 20]. Computational data analysis, including density functional theory (DFT) calculations, provides insights into the thermodynamics [21], kinetics [22], quantum mechanical calculations [23], reactivity [24], and structural properties of solvent-sulfur interactions as well as the structural characteristics of solvent-sulfur complexes. These computational techniques can provide insights into reaction mechanisms, energetics, and the optimization of reaction conditions. Overall, the integration of microwave-assisted extraction with solvent-based desulfurization, supported by computational data analysis, represents a promising approach for achieving cleaner diesel fuel and mitigating environmental impact.

## Experimental

### Material

Methyl alcohol pure (Alpha chemical, assay = 99.9%), acetonitrile (BDH laboratory supplies, assay = 99.7%), ethyl acetoacetate (Sigma Aldrich, assay = 99.0), diesel fuel fraction was supplied by the Suez oil petroleum company.

### Microwave-assisted extractive desulfurization process

**Fig. 1:**
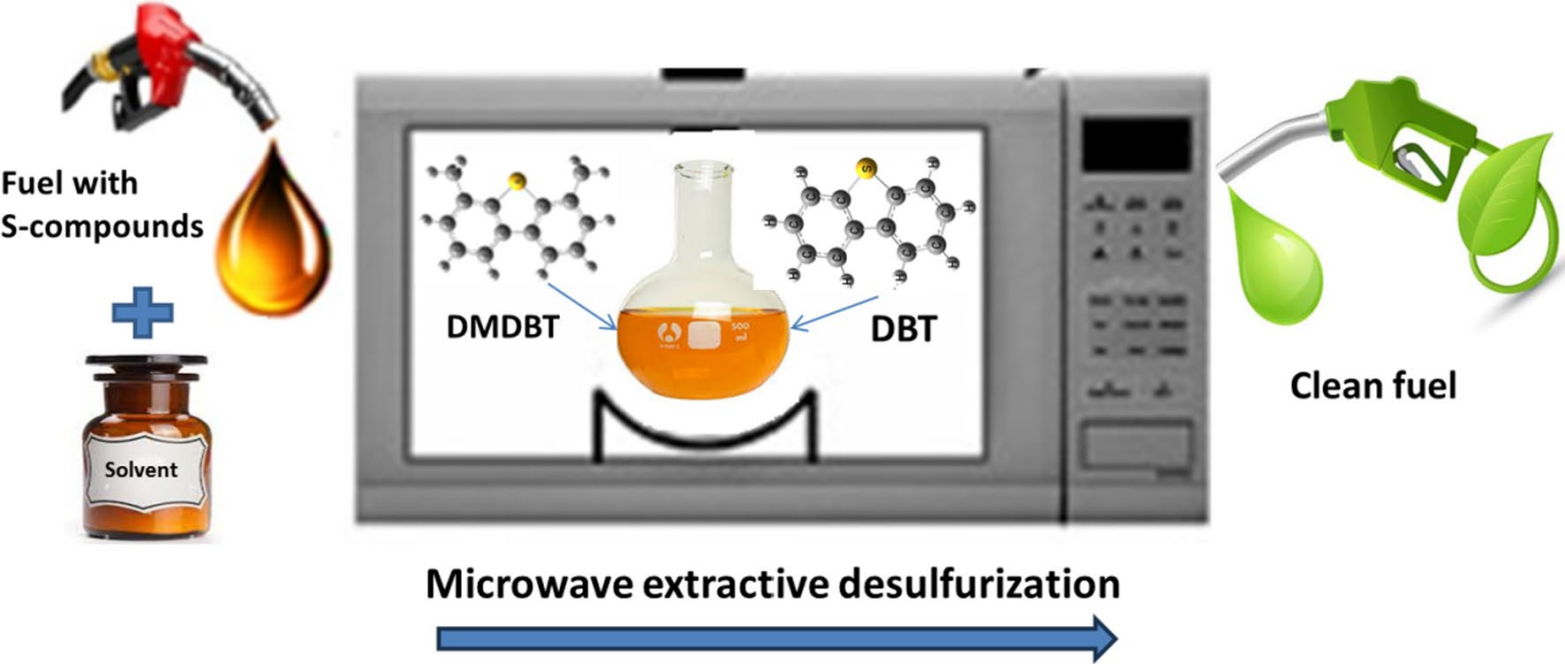
Experimental steps of microwave-assisted extractive desulfurization process

The required amount of solvent (methanol, acetonitrile and ethyl acetoacetate) was added to a 20 ml of the feedstock in the extractor which present inside the microwave in a predefined solvent to feed ratio (v/v) (1:1, 2:1, 3:1 and 5:1) for different time (1, 2, 3, 5,7 and 10 min) and different power (90, 180, and 270 W) with mechanical stirring, after that time the solution left to settle for 10 min, and then separate the raffinate phase. Through many washing times with hot distilled water, the solvent was removed from the raffinate phase. Next, the raffinate was dried over calcium chloride that had been anhydrous. Through distillation at low pressure, the solvent was extracted from the extract phase and subsequently regenerated for future use. Figure 1 represents the stages of microwave-assisted extractive desulfurization process.

## Computational details

Density functional theory (DFT) calculations at the hybrid Functional Becke, 3-parameter, Lee–Yang–Parr (B3LYB) method with 6-311++g(d,2p) basis sets have been applied for geometry optimization and frequency calculations to investigate the strength, the interaction energy, type and the nature of the interaction among dibenzothiophene (DBT) and dimethyl dibenzothiophene (DMDBT) with methanol, acetonitrile and ethyl acetoacetate in gas phase. All calculations were done using the GAUSSIAN 16 program and data was visualized using GaussView software. The interaction energy (E_int_) was calculated as the following:

$$E_{int} = E_{complex} - E_{solvent} - E_{Sulfur} ,$$

where E_complex_, E_solvent_ and E_sulfer_ refer to energy of the solvent binding to the compound containing sulfur, the energy of the solvent and the energy of the compound containing sulfur.

## Characterization

The raffinate of each run (treated diesel fuel) was characterized by determine the density (ASTM D 4052), refractive index (ASTM D542) and sulfur content (ASTM D 4294) using energy dispersive X-ray fluorescence spectrometry.

## Results and discussions

This study’s primary objective is to remove sulfur compounds from diesel fuel. The most crucial step in the processes involved in making diesel fuel is thought to be the extraction of these compounds. This phase is required to enhance the final properties of the diesel fuel that is generated. Fuels are desulfurized selectively by using extracting solvents to remove S-compounds. Traditionally, pyrrolidones, dimethyl sulfoxide, acetonitrile, and dimethylformamide have been the primary solvents utilized as extractants. The process of extractive desulfurization is carried out in relatively mild circumstances and does not require hydrogen or catalysts. Furthermore, sulfur compounds can be removed selectively using EDS without lowering diesel fuel quality. In this study, methanol, acetonitrile, and ethyl acetoacetate were the three distinct solvents used in a microwave-assisted extraction procedure to remove S-compounds. For the past few decades, researchers have examined the application of traditional solvent extraction in this particular subject. However, in order to investigate the additional advantages of using microwave assisted extraction with various solvents generally and with ethyl acetoacetate specifically [11, 25], as well as how we can investigate the advantages of using ethyl acetoacetate for the first time in the sulfur removal process, performing the later extraction technique was necessary in this work. It was crucial to ascertain the properties of the diesel oil (feedstock) used in this investigation before putting the suggested extraction strategies into practice. The physicochemical characteristics of the raw feedstock (diesel oil) are listed in Table 1.

**Table 1:** Physical characteristics diesel oil

Characteristics	Feedstock
Refractive index at 20 °C	1.4650
Density at 20 °C, gm/cm^3^	0.8009
Aromatic content, wt%	16
Sulfur content, ppm	450

### Effect of radiation time

**Fig. 2:**
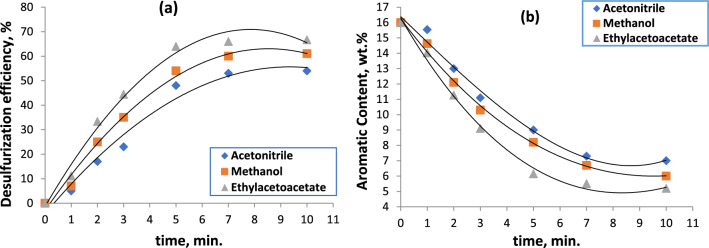
Effect of irradiation time on desulfurization efficiency (**a**) and aromatic content (**b**) through microwave-assisted extractive desulfurization

The impact of varying the duration of microwave radiation exposure on the extraction systems of the feed containing distinct solvents, as well as the influence of solvent composition on the rate of elimination of aromatics and S-compounds, were investigated. In the current stage, a constant solvent to feed ratio of 2/1 and a microwave intensity of 90 W were used. Observations show that a rise in microwave irradiation period from 1 to 10 min was correlated with a consistent rise in desulfurization efficiency; this is accounting for the influence of various solvents, including methanol, ethyl acetoacetate, and acetonitrile. This can be linked to the extraction system being exposed to radiation for a longer period of time (7 min) which increased the rates at which S-compounds and aromatics were removed (Fig. 2a, b).

However, utilizing microwave radiation for duration of one minute did not clearly show any variations in the desulfurization efficacy. There were slight variations in the sulfur and aromatic levels. This is related to the solvent molecules’ produced dipoles not having enough time to interact with the feedstock’s sulfur and aromatic structures. It is evident that a constant increase in desulfurization efficiency was correlated with the microwave radiation time increase from one to seven minutes. After extending the radiation time from 7 to 10 min, a practically constant state in the desulfurization efficiency values was observed.

The two reasons for the enhanced ability of the various solvents to remove sulfur and aromatic compounds using the microwave-assisted extraction technique are as follows: first, as the procedure time increased, the solvent molecules’ increased absorbance of microwave radiations could stimulate the electronic system within the solvent’s structure more and more, forming a enormous number of dipoles on the solvent molecules and enhancing the static interaction among the solvent molecules and the sulfur and aromatic compounds in the feedstock. Consequently, an increase in the amount of removed sulfur and aromatic compounds could be achieved.

Because each solvent has a different chemical structure, the percent of sulfur and aromatic compound removal varies [26]. For example, acetonitrile has one nitrogen atom, while methanol has one oxygen atom. Because oxygen has a higher electronegativity than nitrogen, methanol is more polar than acetonitrile when microwave radiation is present, increasing its efficiency and selectivity for the poly aromatic compounds that are associated with sulfur atoms. In contrast, ethyl acetoacetate has three oxygen atoms, increasing its selectivity and efficiency when using microwave radiation to remove sulfur and aromatic compounds. Thus, it is evident to us that the ideal solvent for this step is ethyl acetoacetate.

The second explanation is that the extraction system’s temperature rose as a result of longer exposure to microwave radiation. Increased removal of sulfur and aromatic compounds may be possible since rising extraction temperatures are typically accompanied by rising solvent powers.

### Effect of solvent to feed ratio (S/F)

**Fig. 3:**
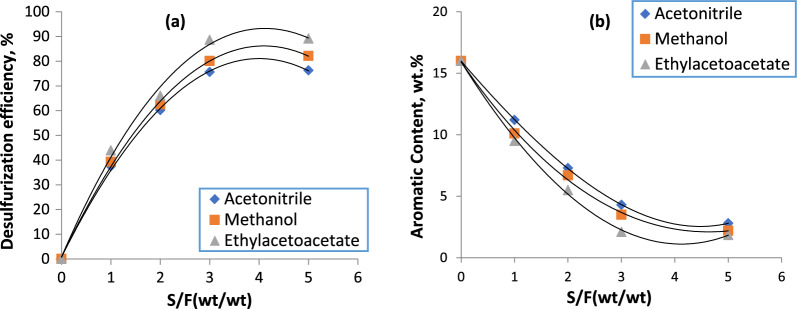
Effect of solvent to feed ratio on desulfurization efficiency (**a**) and aromatic content (**b**) through microwave-assisted extractive desulfurization

Following the previous step’s determination of the optimal radiation period of 7 min, this time was used to investigate the impact of the S/F ratio on the quality of the desulfurization and dearomatization processes. The acquired data demonstrate that increasing the solvent feed ratio can both raise desulfurization efficiency (Fig. 3a) and lower aromatic content values (Fig. 3b). As the solvent feed ratio was increased from 1:1 to 3:1, a sharp increase in the desulfurization efficiency values along with a decrease in the aromatic content of all of them could be noticed, in agreement with Fig. 3, which depicts the decline gradient in the percentages of aromatic content and increasing desulfurization efficiency (Fig. 3a, b).

After that, declines that were almost slower occurred when the S/F was increased to 5:1. This can be explained by the fact that when the S/F was first raised to 3:1, the selectivity and capacity of the solvents were also altered. But if S/F is increased even further to 5:1, this might significantly boost the solvent’s power, which was already diminished by a drop in selectivity. As a result, there had been a modest change in the tendency of all solvents to remove aromatics and sulfur compounds. As a result, the desulfurization efficiency increased less (Fig. 3a, b). The S/F of 3:1 was determined to be the ideal condition for carrying out the extraction process while subjected to microwave radiation by taking the economic concept into account. Furthermore, compared to a ratio of 3:1, a substantially higher amount of energy is needed to recover the solvent following the extraction process when the S/F value is equal to 5. This may also indicate that the solvent feed ratio indicated later is advantageous for using in such a process.

### Effect of radiation intensity

**Fig. 4:**
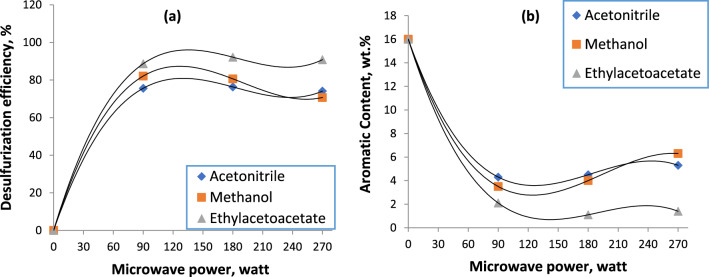
Effect of microwave intensity variation on desulfurization efficiency (**a**) and aromatic content (**b**) through microwave-assisted extractive desulfurization

The study examined the impact of varied microwave intensity on the extractive desulfurization process and the resultant decrease in the aromatic content. Three different microwave radiations powers 90, 180, and 270 W were employed during this phase. When the radiation intensity was raised from 90 to 180 W, the concentration of sulfur in the instance of ethyl acetoacetate decreased and the increase in the percentages of aromatic compounds removal brought on by the higher microwave power applied (Fig. 4a, b). There has been a reported increase in the percentages of aromatics removal in general and sulfur-aromatics removal in particular due to the increase in radiation absorbed by the solvent molecules. Consequently, the injected solvent’s dipole moment can rise, which could lead to an improved removal of sulfur compounds. In the case of using methanol and acetonitrile, there was a decrease in the efficiency of the sulfur removal process. This can be explained because the boiling point for both methanol and acetonitrile is 36.7 and 82, respectively. As the intensity of microwave radiation increases, the temperature increases, this causes a malfunction in the extraction system. It causes the solvents to lose part of their solvent power and selectivity for extraction. We confirmed this when the power of the microwave radiation was increased to 270 W. The efficiency of the process of removing sulfur compounds and also aromatic compounds deteriorated further. This confirms that the use of both methanol and acetonitrile at high intensity radiation Microwave at 180 or 270 W for a time of 7 min is not permissible.

When the intensity of microwave radiation was increased from 180 to 270 W in the presence of ethyl acetoacetate, a decrease was observed in the efficiency of removing sulfur and, consequently, the removal of aromatic compounds. This is explained by the fact that as increasing the radiation absorbed by solvent molecules, the temperature was risen but not exceed to 100 °C. Where, the boiling point of this solvent is 180. 8 °C, so that the solvent will be in a stable state, but the hydrostatic bonds that connect the sulfur compounds to the solvent molecules applicable to separate, which will worsen and hinder the extraction process. Thus, the best solvent can be used in the process. Extraction of sulfur compounds is ethyl acetoacetate at a microwave intensity of 180 W.

The corresponding energy consumptions of conventional and microwave assisted extraction techniques were calculated according to the following equation, as reported by Theraja [27].

$${\text{Energy}}\;{\text{consumption}}\;\left( {{\text{KWh}}} \right) = {\text{Power}}\;{\text{consumed}}\;\left( {{\text{KW}}} \right) \times {\text{Time}}\;\left( {\text{h}} \right).$$

It could be detected that the energy consumption by the conventional method equal 1875 KWh instead of being 21 KWh for microwave assisted extraction process. This, in turn, could demonstrate the massive reductions in energy consumption using the presented MAE extraction technique in this study.

To unravel the mechanism of extractive desulfurization by acetonitrile, methanol, and ethyl acetoacetate, a computational study was conducted. This study included structural investigations, and interaction energy analysis to examine the type and nature of the interactions between dibenzothiophene (DBT) and dimethyl dibenzothiophene (DMDBT) with acetonitrile, methanol, and ethyl acetoacetate.

**Fig. 5:**
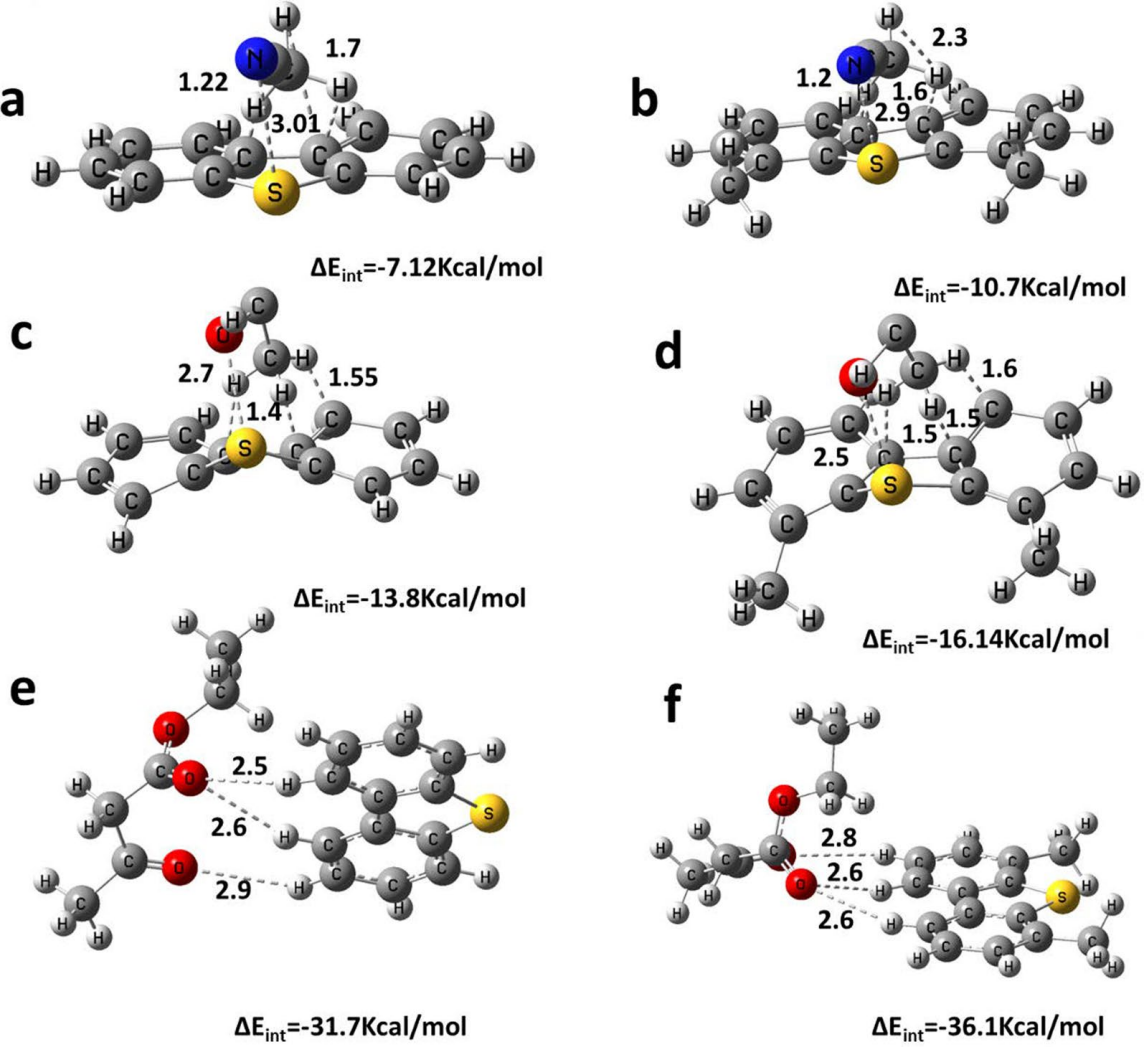
Optimized geometries and calculated interaction energies of **a** DBT with acetonitrile **b** DMDBT with acetonitrile **c** DBT with methanol **d** DMDBT with methanol, **e** DBT with ethyl acetoacetate, **f** DMDBT with ethyl acetoacetate. Dotted lines showing intermolecular hydrogen bonding

The optimized geometries of DBT and DMDBT and their complexes with acetonitrile, methanol, and ethyl acetoacetate are presented in Fig. 5. Their calculated interaction energies were also determined. Acetonitrile, methanol, and ethyl acetoacetate form robust interactions with DBT and DMDBT. Supramolecular chemistry heavily relies on non-covalent weak interactions, such as π–π interactions, weak hydrogen bonding, electrostatic interactions, and hydrophobic–lipophilic interactions. These non-covalent interactions might be the most effective mechanisms for extracting sulfur-containing compounds from fuel.

In acetonitrile, the presence of an electronegative nitrogen atom draws electrons from carbon atoms, thereby growing the positive charge on the hydrogen atoms of the methyl group, making it more effective as a “π–hydrogen bond” donor. The three hydrogens in the methyl group form three bonds with carbon in both DBT and DMDBT (C21–H23$\cdots$C3, C21–H24$\cdots$C2, and C21–H25$\cdots$C13), with bond distances of 1.22, 1.7, and 1.7 Å for DBT, and 1.6, 1.6, and 2.3 Å for DMDBT.

In methanol, the more electronegative oxygen atom draws electrons more strongly than nitrogen in acetonitrile, resulting in shorter C–H bonds. The three hydrogens in the methyl group form three bonds with carbon in both DBT and DMDBT (C21–H23$\cdots$C3, C21–H24$\cdots$C2, and C21–H25$\cdots$C13), with bond distances of 1.4, 1.4, and 1.55 Å for DBT, and 1.5, 1.5, and 1.6 Å for DMDBT. Additionally, oxygen forms stronger bonds with the sulfur atom of DBT and DMDBT, with shorter bond lengths of 2.7 Å and 2.5 Å, respectively, compared to nitrogen in acetonitrile.

For ethyl acetoacetate, the presence of three oxygen atoms increases electronegativity and robustly attracts electrons. The interaction energies for acetonitrile-DBT, acetonitrile-DMDBT, methanol-DBT, methanol-DMDBT, ethyl acetoacetate-DBT, and ethyl acetoacetate-DMDBT are − 7.12, − 10.7, − 13.8, − 16.14, − 31.7, and − 36.1 kcal/mol, respectively, in gas-phase calculations. A donor–acceptor interaction among methanol and DBT or DMDBT, or between ethyl acetoacetate and DBT or DMDBT, was observed, indicating charge transfer among the lone pair of the sulfur atom of DBT or DMDBT and the hydroxyl group of methanol or the carbonyl and carboxyl groups of ethyl acetoacetate. Smaller values of donor–acceptor interactions were noticed among the lone pair of the sulfur atom and the nitrogen atom in acetonitrile.

The dipole moments of DBT and DMDBT in the gas phase are 0.768 D and 0.0367 D, respectively. In addition to hydrogen bonding, O–H and the O–S bonds, the higher polarity of DMDBT compared to DBT could account for the higher interaction energies of methanol ⊃ DMDBT versus methanol ⊃ DBT and ethyl acetoacetate ⊃ DMDBT versus ethyl acetoacetate ⊃ DBT. The polarizability was calculated to be 188.8 and 160.9 a.u. for DMDBT and DBT, respectively.

## Conclusions

This study introduces an innovative approach for desulfurizing commercial diesel fuel. The reactivity of various organic solvents in the microwave-assisted extractive desulfurization and dearomatization technique is being investigated. Ensuring a sustainable solvent (methanol, acetonitrile, or ethyl acetoacetate) is one of the main goals. Another is investigating the effects of the solvent/feed ratio, microwave intensity, and irradiation period. The results showed that ethyl acetoacetate is the most appropriate solvent with microwave assisted extractive desulfurization approach and this is attributed to its polarity. Additionally, the most efficient conditions which resulting in a substantial increase in desulfurization efficiency about ≈ 92% are the irradiation time is 7 min, the solvent feed ratio is 3:1 and the microwave intensity is 180 W. In addition, a computational study at the B3LYB/6-311++g(d,2p) has been performed on the interaction between dibenzothiophene (DBT) or dimethyl dibenzothiophene (DMDBT) and the different organic solvents. The results not only unveil the interaction between them but they also support our experimental data for the microwave assisted desulfurization process of the fuel using the different organic solvents. These discoveries have the potential to advance eco-friendly technologies and support international initiatives to lower air pollution and advance transportation-related sustainable energy solutions.

## Data Availability

The datasets used and/or analyzed during the current study are available from the corresponding author on reasonable request.

## Abbreviations

DBT: Dibenzothiophene
DMDBT: Dimethyl dibenzothiophene
US-EPA: United States Environmental Protection Agency
MAE: Microwave assisted extraction
DFT: Density functional theory
B3LYB: Becke, 3-parameter, Lee–Yang–Parr method
EDS: Extractive desulfurization
S/F: Solvent to feed
